# Brief Report From the 4th International Meeting on Bone Marrow Adiposity (BMA2018)

**DOI:** 10.3389/fendo.2019.00691

**Published:** 2019-10-18

**Authors:** Guillaume Penel, Greet Kerckhofs, Christophe Chauveau

**Affiliations:** ^1^Inflammatory Bone Diseases Lab, Univ. Littoral Côte d'Opale, Boulogne-Sur-Mer, and Univ. Lille, Lille, and CHU Lille, Lille, France; ^2^Biomechanics Lab, Institute of Mechanics, Materials and Civil Engineering, UCLouvain, Louvain-la-Neuve, Belgium; ^3^Department Materials Engineering, KU Leuven, Leuven, Belgium

**Keywords:** bone marrow adiposity, bone marrow adipocytes, bone marrow fat, yellow marrow, bone metabolism, bone marrow adiposity society (BMAS)

## Abstract

The 4th International Meeting on Bone Marrow Adiposity (BMA2018) was hosted at the premises of the Regional Government of *Hauts de France* in Lille, from August 29th to August 31st 2018. This congress brought together physicians and scientists working on rheumatology and bone biology, oncology, hematology, endocrinology, and metabolic diseases, all interested in bone marrow adiposity. They shared their opinions, hypothesis, and original results. Six invited keynotes were given by S. Badr, B.C.J. van der Eerden, M.J. Moreno Aliaga, O. Naveiras, C.J. Rosen, and A.V. Schwartz. Twenty-one short talks were also given. This report briefly summarizes the scientific content of the meeting and the progress of the working groups of the BMA Society (http://bma-society.org/).

## Introduction

The 4th International congress on Bone Marrow Adiposity (BMA) was hosted at the premises of the Regional Government of *Hauts de France* in Lille, From August 29th to August 31st 2018 (https://bma2018.sciencesconf.org). The meeting was organized by C. Chauveau and G. Penel from the laboratory *Pathophysiology of Inflammatory Bone Diseases* EA4490 (currently becoming *Marrow Adiposity and Bone Lab*) located in Lille (University of Lille) and Boulogne sur Mer (Littoral Côte d'Opale University), with the collaboration of G. Kerckhofs from the Skeletal Biology and Engineering Research Center, KU Leuven, and the Institute of Mechanics Materials and Civil Engineering, UCLouvain, Belgium. Attendees were coming from 13 European countries, Canada and USA.

Three annual meetings were previously organized in Lille, France (https://bma2015.sciencesconf.org) (1); in Rotterdam, the Netherlands (https://adiposity.sciencesconf.org/) (2); and in Lausanne, Switzerland (https://bma2017.sciencesconf.org) (3). The fourth annual meeting, once again organized in Lille, France, brought together physicians and scientists working on rheumatology and bone biology, oncology, hematology, endocrinology, and metabolic diseases, all interested in BMA. The meeting was organized in the continuity of the previous ones, with the same purpose of facilitating exchanges between members of the emerging scientific community interested in BMA. To reflect the diversity of the attendees, oral communications were organized in five sessions: bone marrow adipocyte (BMAd) biology, BMA and clinical translation, BMA imaging, BMA in hematology and cancer, and finally BMA and metabolism. Details on the sessions and the contributors are given in Appendix A (Supplementary Material). For the first time, *Meet the Professor* sessions were organized with the precious involvement of C.J. Rosen and A.V. Schwartz. Finally, each working group, dedicated to *nomenclature, methodologies, biobank, public engagement, data repositories*, and *sponsorship* met to start or continue its work.

The submitted abstracts were ranked by all members of the Scientific Board of the BMA Society (BMAS), leading to 21 oral presentations and eight poster presentations. Two junior presentations (by M. Tencerova and A. Lovdel) were awarded by the Scientific Board of the BMAS by a free registration for the next BMA congress in Odense, Denmark.

In the present report, data unpublished at the time of the congress were highlighted by the symbol §.

## Scientific Communications and Debates

### Bone Marrow Adipocyte Biology

Data on the biology of BMAds remain rare and far between. Moreover, some of these data appear to be contradictory. This situation justified a session dedicated to the biology of BMAds including two invited talks given by Prof. C.J. Rosen and Prof. B.C.J. van der Eerden. Based on relevant and remarkable data from the literature and their own work, speakers highlighted the specificity of BMAds related to their location in the skeleton and the hematopoietic niche, their endocrine, paracrine and autocrine functions, and the unique regulation of BMA. The main points presented by C.J. Rosen were: (i) links between PGC1α expression and BMA in mice depending on the bone analyzed (4), (ii) the effect of PTH on BMAd size, perhaps through stimulation of lipolysis (5), (iii) the hypothetic involvement of differentiation and dedifferentiation processes allowing changes between adipocyte and pre-adipocyte states (6), and (iv) links between mitophagy in stromal cell differentiation and their activity or ability to differentiate §. B.C.J. van der Eerden introduced the use of bioinformatics to study skeletal stem cell adipogenesis pathway. His study of the changes that occur in gene expression during the very early moments after induction revealed the first temporality of the processes that lead to adipocyte differentiation. This wide approach allowed the use of Connectivity Map (CMap) leading to the identification of new factors like parbendazole that are highly involved in adipocyte or osteoblast differentiation (7).

Other presented data gave more information on the nature of BMAds. In their study, E.L. Scheller and C.S. Craft used 3D electron microscopy to show that these cells have an extensive mitochondrial network (8). They are capable of adrenergic-induced lipid droplet remodeling but neither of UCP1 expression nor thermogenesis *in vivo* (9).

Relationships between BMAds and RANKL were also discussed in two studies. Indeed, N. Bravenboer showed that BMAds display changing activities like expression of RANKL after ovariectomy §. E. Douni revealed that transgenic overexpression of RANKL is associated in mice with high BMA and high bone resorption. Moreover, the same study showed that the inhibition of osteoclastogenesis is associated with low BMA §. R. Labella demonstrated high bone resorption and high osteoclastogenesis in female mice with a gain of function mutation of Gsα expressed under the control of an AdipoQ promoter, suggesting that BMAds may affect the bone/bone marrow microenvironment through the Gsα/cAMP signaling pathway §. Finally, G. Frangi pointed out links between the inactivation of the sodium-phosphate co-transporter, recently shown to be deleterious for ossification and bone quality (10), and BMA. Interestingly, the induced BMA increase is not associated with changes in plasma levels of adiponectin §.

### BMA and Clinical Translation

Prof. A.V. Schwartz gave an invited lecture on the clinical determinants of BMA. While it was shown that BMA is under the control of gonadal and pituitary hormones in rodents, conclusive studies on the changes and differences in BMA that occur between men and women, during life and during menstrual cycle, remain scarce (11). Clinical and translational studies suggest that, depending on the situation or protocol, estradiol and testosterone plasma levels, or treatments with these hormones could be associated with a slightly low vertebral BMA in men and women for both kind of hormone (12, 13).

Data from two observational studies on several hundreds of subjects from the AGES—Reykjavik cohort were presented. The first one, presented by G. Woods, identified high vertebral BMA as a predictor of greater reductions in bone quality in spine, hip and femoral neck of older women §. The second one, presented by A. Veldhuis-Vlug, demonstrated an association between FSH and greater vertebral BMA in older women. Interestingly, for both studies no association was found in older men §.

Data from two interventional studies were also presented. A. Velduis-Vlug, starting from the positive effects of PTH treatment on bone from mice associated with decreased BMA, showed a trend toward lower BMA after treatment with Abaloparatide or Teriparatide in postmenopausal women (Abaloparatide-SC Comparator Trial In Vertebral Endpoints -*Active-* trial) with osteoporosis with beneficial effects on bone §. P.H. Bisschop pointed out the bariatric surgery (Roux-en-Y gastric bypass)-induced decrease in vertebral BMA of non-diabetic post-menopausal women. Compared with other studies, these data lead to suppose different effects of RYGB on BMA and bone depending on menopausal and diabetic statuses §.

### BMA Imaging

Exploring *in vivo* bone marrow adiposity (BMA) using clinical imaging tools remains challenging. It is also of crucial interest to bring clinical and basic research closer together, as this could lead to relevant therapeutic solutions. In his invited lecture, S. Badr proposed magnetic resonance imaging (MRI) as the current best available technique to qualitatively and quantitatively assess BMA, especially through single-voxel proton spectroscopy and chemical shift encoding-based water fat imaging. However, interfaces between trabecular bone and BMA foster a local magnetic inhomogeneity that must be taken into consideration to provide accurate measurements (14). He proposed that future works might aim to extend the two main clinically available quantitative parameters—the proton-density fat fraction and lipid unsaturation level—to other imaging features to best depict BMA.

Additionally, two studies that illustrated what could be expected of MRI tools were presented. N. Sollmann investigated the relationship between vertebral BMA and paraspinal muscle fat composition, two parameters shown to be altered in various diseases. Interestingly, postmenopausal but not premenopausal women display high and correlated levels of fat in the two tissues (15). E. Burian presented a second study designed to better characterize BMA, and particularly its spatial heterogeneity. This feasibility study was driven on healthy pre- and postmenopausal women, using chemical shift encoding-based water-fat magnetic resonance imaging. Data from this study led to the validation of the approach and allowed to conclude to an increased bone marrow heterogeneity in lumbar spine of postmenopausal women (16).

### BMA in Hematology and Cancer

The close location of BMAds and hematopoietic stem cells (HSCs) in the bone marrow, as well as results from *in vitro* experiments, support the hypothesis of direct interactions between these two cell types (17). During her invited lecture, Prof. O. Naveiras highlighted the rapid transition from red to yellow marrow within 1–3 weeks after chemotherapy, radiotherapy, or even food restriction and in some situations the fast reversion to red marrow. Also, the differences between environments of endosteal and perivascular HSC were taken into account, and the relationships between BMAds and HSCs seem to be complex because BMAds appear to compromise HSC-derived progenitors but not primitive HSC functions. Moreover, BMAds and preadipocytes seem to act differently on hematopoiesis.

Several studies highlighting these relationships were presented. With the ambition to accelerate the red-to yellow-to red transition after irradiation-induced bone marrow aplasia, S. Rojas-Sutterlin presented a first study that characterized changes of the stromal cell compartment in this situation, and identified phenotypic populations with differential kinetics. The adipocyte differentiation axis was particularly studied and associated with hematopoietic recovery §.

D. Mattiucci showed the results of a second study, also dealing with this relationship but in a caloric restriction situation. He concluded that adiponectin is mainly produced by BMAds in this situation and is not directly acting on bone marrow HSCs, but it contributes to the immunomodulatory effects of caloric restriction §.

M. Reagan studied the involvement of BMAds in the efficacy of antimyeloma treatments with dexamethasone. She showed that BMAds or their conditioned media support MM1S myeloma cells drug resistance, possibly through the action of IL-6. Moreover, treatment with anti-sclerostin antibody to decrease BMA *in vivo* lowers the resistance properties of myeloma cells §.

### BMA and Metabolism

It is well-known that obesity and associated disorders are related with dysfunctional white adipose tissue (WAT) metabolism and a secretory pattern. During her invited lecture, Prof. M.J. Moreno Aliaga wondered whether brown adipose tissue (BAT) could be an anti-obesity and anti-diabetic tissue. She highlighted the therapeutical potential of some of the specialized pro-resolving lipid mediators (SPMs) such as maresin1, on dysfunctional adipose tissues (18). In ob/ob and diet-induced obesity mice, this SPM induces beneficial effects on WAT, activates BAT, and decreases insulinemia (19).

Six studies were presented during this session. K. Suchacki showed that adipocytes from the bone marrow and the WAT in human but also from rabbits are molecularly and functionally distinct. She demonstrated that insulin does not induce an increase in glucose uptake in most of the bone marrow cavities, but interestingly this result is depending on anatomic localization §.

M. Tencerova showed results on high fat diet mice suggesting that the diet induces insulin resistance in mesenchymal stem cells from subcutaneous adipose tissue but not in skeletal stem cells from bone marrow (20). This study also showed an increased insulin signaling in BMSCs from obese patients compared to those from lean subjects, hypothesizing that these data reflect an evolutionary conserved mechanism for bone as energy storage organ §.

Interestingly, in the postmenopausal state, also associated with metabolic alterations, S. Lucas demonstrated that BMAds show an evolving phenotype. In this context, or under high glucose exposure, BMAds express pro-osteoclastic, and/or anti-osteoblastic factors suggesting that they could contribute to the bone loss of postmenopausal osteoporosis §.

C.S. Craft enlarged the field of thinking by including the nervous system as a possible regulator of BMA. Indeed, her study showed that neuropathy-resistant mice are protected against type-1diabetes-induced neuropathy, bone marrow fat expansion, and bone alterations §.

P. Boroumand's study focused on the relationship between obesity-induced inflammation and bone marrow. Notably, she wondered whether increased BMA favors the development of inflammatory monocytes in this context. *In vivo* and *in vitro* results suggest that the high fat diet-induced changes in BMAd functions could be directly responsible for the activation of monocytes in the bone marrow §.

A last study from A. Lodvel on the involvement of glucocorticoids on caloric restriction-induced BMA increase revealed that in this context BMAT expansion is not sufficient for trabecular bone loss and that caloric restriction-induced alterations of bone and bone marrow are sex-dependent in mice. Moreover, the knockout of 11b-hydroxysteroid dehydrogenase type 1 protects male mice but not female mice from caloric restriction-induced increase in BMA §.

## Working Groups

### Nomenclature

Among the most developed groups, the working group for *Nomenclature* (Chairman: W.P. Cawthorn) has been working for almost 1 year to identify the terminology that has been used in the BMA field, and to propose a unified nomenclature system that will provide clarity and consensus as research into BMA continues to increase. The *Nomenclature* working group has pursued this work on the basis of the literature and the wide expertise of its members. During its meeting session at BMA2018, this working group validated propositions on 28 terms that have been used in the BMA research field, deciding which of these terms to use, and which to stop using. For those terms recommend to use, an exact meaning was also proposed. Finally, the group agreed to start the writing of a white paper on this subject. These recommendations are proposed as a review article in the present issue on BMA.

### Methodologies

The working group *Methodologies* (Chairwomen: O. Naveiras and A. Veldhuis-Vlug) is preparing a review paper on the existing methodologies to characterize BMA. The working group members are all active researchers in the field of BMA, both clinical and preclinical. The working group members searched the literature for articles describing the investigation of BMA and discussed the results during personal and telephone conferences. The consensus opinion, both based on the review of the literature and on expert opinion, will be discussed in the review. The following topics will be tackled: (1) cell isolation, culture, differentiation and *in vitro* modulation of primary bone marrow adipocytes and adipocytic precursors, (2) histomorphometry of bone marrow adipocytes in 2D and 3D, (3) imaging of BMA *in vivo* with magnetic resonance imaging, (4) imaging of BMA *ex vivo* with contrast enhanced computed tomography, (5) *in vitro* microscopy, and (6) modulation of BMA in animal models.

### Biobank

The BMAS working group on *Biobank* (Chairman: B.J.C. van der Eerden) has discussed how to proceed with its ambition to generate standardized approaches toward isolation, characterization and long-term storage of tissues/cells and data related to BMA. Additional aspects of biobanking that the working group will scrutinize are privacy regulations regarding participants/patients, data protection, and ethical guidelines. Obviously, within an international community focusing on BMA with a desire to unify methodologies and guidelines, challenges will emerge, and which will be the focus of the working group.

### Public Engagement

Chair position for the working group *Public engagement* was transferred from E.L. Scheller to C.S. Craft. This working group proposed to assist E.L. Scheller in planning 2020 Seattle BMAS meeting. Members of this group would like to set up a LinkedIn account for BMAS similar to ASBMR's page. We also suggest adding a lab website link to each BMAS member name on the BMAS website.

### Data Repositories

A first meeting of the working group *Data repositories* (Chairman: A. van Wijnen) allowed discussions on the objective of sharing data and on how to obtain omics data suitable for later comparisons.

### Sponsorship

Chair position for the working group *Sponsorship* was transferred from G. Penel to P.H. Bisschop. Among the items discussed during this first meeting of the working group a standardization of the contracts with the sponsors of the congresses was proposed on the basis of those used for the current meeting. The presentation of the sponsors on the websites (congress and society) was identified as a crucial point.

## Concluding Remarks and Perspectives

This congress confirmed the dynamism of this young field of research. Indeed, numerous investigators showed their results related to all the fields of research interested in BMA (rheumatology and bone biology, oncology, hematology, endocrinology, and metabolic diseases). Moreover, several of the presented studies have been published since the congress (5, 8–10, 14, 15) or just before (4, 6, 13, 16–18, 20).

For the first time, each working group within the BMA Society (BMAS) met to work on its specific topic, resulting in the submission of two white papers. A long term work was initiated by the other working groups.

Also, the organization of the 2020 Seattle BMA congress was strengthened. Indeed, the young and dynamic BMAS discussed the organization of future BMA congresses. BMA 2019 will be organized in Odense (Denmark), whereas BMA2020 will be affiliated to the annual congress of the American Society for Bone and Mineral Research (ASBMR) in Seattle (USA).

As future perspective, the authors believe that the BMAS has to play a crucial role in holding this multidisciplinary community together, and in stimulating close interactions between the players in this field of research. Indeed, it is research at the forefront of scientific and technological development and knowledge, as confirmed by the presentations given during BMA 2018 congress, which brought a large overview on this tissue and demonstrated its involvement in numerous physiological functions and pathologies. One of the most interesting points was that BMAds seem to be adaptive and their activities depend on the context. Thus, a multidisciplinary approach seems essential to allow a rapid and certain progression of knowledge of this tissue and the use of this knowledge for therapeutic purposes. Additionally, we are convinced that the development of clinical imaging tools for routine assessment of BMA will be a challenging but decisive step.

## Author Contributions

All authors listed have made a substantial, direct and intellectual contribution to the writing of the congress report and approved it for publication.

### Conflict of Interest

Authors wish to thank Lilly, Linsey Tec, UCB and Dutscher as sponsor companies who supported the organization of the congress. The funders had no role in study design, data collection and analysis, decision to publish, or preparation of the manuscript.
