# Supplementary material for: Brief Report From the 4th International Meeting on Bone Marrow Adiposity (BMA2018)
Source: Front Endocrinol (Lausanne). 2019 Oct 18;10:691. doi: 10.3389/fendo.2019.00691 (PMC6813723; doi:10.3389/fendo.2019.00691)
Supplement: Supplementary file 1 [file Data_Sheet_1.PDF]

Appendix A: Scientific sessions and contributors

Session 1: Bone marrow adipocyte biology

Bone Marrow Adiposity in 2018: Changes and Challenges - **CJ Rosen** (Maine Medical Center Research Institute, Scarborough, Maine, USA)

Novel approaches to delineate skeletal stem cell commitment towards adipocytes – **BCJ van der Eerden** (Erasmus MC, Rotterdam, The Netherlands)

Bone marrow adipose tissue: white, brown or beige? - **E Scheller** (Washington University, Division of Bone and Mineral Diseases, Saint Louis, MO, USA)

Ovariectomy increases RANKL protein expression in bone marrow adipocytes of C3H/HeJ mice - **N Bravenboer** (Amsterdam University Medical Centers, Amsterdam and Leiden University Medical Center Leiden, The Netherlands)

Bone marrow adiposity in modeled osteoporosis - **E Douni** (Biomedical Sciences Research Center "Alexender Fleming", Athens, and Laboratory of Genetics, Agricultural University of Athens, Greece)

AdipoQ-cre;Rosa26R201C mice develop a complex skeletal phenotype with different features in cMAT and rMAT regions - **R Labella** (Department of Molecular Medicine, Sapienza University of Rome, Italy)

Role of pit2 in bone and adipose tissues inter-communication? **G Frangi** (Inserm U1229, RMeS STEP group Skeletal physiopathology and joint regenerative medicine, Université de Nantes, CHU Nantes, France)

Session 2: Bone marrow adiposity and clinical translation

Gonadal and pituitary control of BMAT - **AV Schwartz** (Department of Epidemiology and Biostatistics, University of California, San Francisco, USA)

Effect of Abaloparatide and Teriparatide on marrow adipose tissue in postmenopausal osteoporosis - **A Veldhuis-Vlug** (Maine Medical Center Research Institute, USA)

Effect of Roux-en-Y gastric bypass on bone marrow adipose tissue and bone mineral density in non-diabetic postmenopausal women - **P Bisschop** (Amsterdam University Medical Centers, Amsterdam, The Netherlands)

Greater Bone Marrow Adiposity Predicts Loss of Spine Compressive Strength and Trabecular Bone in Postmenopausal Women - **G Woods** (University of California, San Diego and VA San Diego Medical Center San Diego, USA)

FSH is positively associated with vertebral bone marrow adiposity in postmenopausal women from the AGES-Reykjavik cohort - **A Veldhuis-Vlug** (Maine Medical Center Research Institute, USA)

Session 3: Bone marrow adiposity imaging

Imaging bone marrow adiposity in human studies: What is inside my toolbox? A radiologist's point of view. **S Badr** (Divison of Radiology and Imaging, CHU Lille, France)

Associations between lumbar vertebral bone marrow and paraspinal muscle fat compositions – an investigation by chemical shift-encoding based water-fat MRI. **N Sollmann** (Dept Diagnostic and Interventional Neuroradiology, Kilinikum rechts der Isar, Tehcnische Universität München, Germany)

Texture analysis of vertebral bone marrow using chemical shift encoding-based water fat MRI a feasibility study. **E Burian** (Dept Diagnostic and Interventional Neuroradiology, Kilinikum rechts der Isar, Tehcnische Universität München, Germany)

Session 4: Bone marrow adiposity in hematology and cancer

Bone Marrow Adiposity: A haematologist perspective. **O Naveiras** (Laboratory of Regenerative Hematopoiesis, ISREC & Institute of Bioengineering, EPFL, Lausanne, Switzerland)

Targeting BMAT in vivo and in vitro increases efficacy of dexamethasone. **M Reagan** (Maine Medical Center Research Institute, Scarborough, USA)

Kinetic analysis of mouse stromal cell compartment upon irradiation-induced bone marrow aplasia. **S Rojas-Sutterlin** (Institut Suisse de Recherches Expérimentales sur le Cancer, EPFL, Lausanne, Switzerland)

Caloric Restriction and haematopoiesis: is there a role for adiponectin? **D Mattiucci** (Center for Cardiovascular Science, Queen’s Medical Research Institute, University Edinburgh, UK)

Session 5: Technologies and engineering approaches for assessing bone marrow adiposity

Dysfunctional adipose tissues in obesity and inflammation: physiological and therapeutic role of specialized pro-resolving lipid mediators. **M J Moreno-Aliaga** (Dept Nutrition, Food Science and Physiology, Center for Nutrition Research, University of Navarra, Pamplona, Spain)

Absence of insulin resistance in bone marrow stromal stem cells in obesity- lessons from mice to humans. **M Tencerova** (Dept Molecular Endocrinology, University Southern Denmark and Odense Hospital, Odense, Denmark)

Investigating glucocorticoids as mediators of increased bone marrow adiposity during caloric restriction. **A Lovdel** (Center for Cardiovascular Science, Queen’s Medical Research Institute, University Edinburgh, UK)

Bone Marrow Adipocytes in the post-menopausal state show an evolving phenotype that can contribute to altered bone remodeling. **S Lucas** (Pathophysiology of Inflammatory Bone diseases, University Littoral Côte d’Opale, Boulogne sur Mer, France)

Bone marrow adipose tissue is molecularly and functionally distinct to white and brown adipose tissue. **K Suchacki** (Center for Cardiovascular Science, Queen’s Medical Research Institute, University Edinburgh, UK)

Peripheral neuropathy contributes to diabetes-induced bone fragility and marrow adipose tissue expansion. **C Craft** (Washington University, Division of Bone and Mineral Diseases, Saint Louis, MO, USA)

Influence of obesity and bone marrow adiposity on monocyte development: implications for metabolic disease. **P Boroumand** (University of Toronto, Hospital for Sick Children, Toronto, Canada)
